# A genotype-by-sequencing dataset and identity-by-state matrix of genetic variation in 821 *Pinus radiata* trees from 16 counties

**DOI:** 10.1016/j.dib.2025.112123

**Published:** 2025-10-03

**Authors:** Sarah L. Addison, Luciana L. Mendoza, Megan A. Rúa, Peter W. Clinton, (Global Tree Root Microbiome Consortium), Steve A. Wakelin

**Affiliations:** a Bioeconomy Science Institute, Rotorua 3046, New Zealand; b Island Ecology and Conservation Group (GECI), AC Moctezuma #836, Centro Ensenada, Baja California, Mexico; c Department of Biological Sciences, Wright State University, Dayton, 45435, OH, United States; d Environmental Sciences PhD program, Wright State University, Dayton, 45435, OH, United States; e Bioeconomy Science Institute, Riccarton, Christchurch 8440, New Zealand

**Keywords:** Conservation, Forestry, Genetic gain, Population ecology

## Abstract

*Pinus radiata* D. Don is one of the most widely planted conifer species globally. While it holds major commercial importance as a softwood plantation tree, its native populations are endangered, making it a species of high conservation value. Here, we present a comprehensive dataset of genetic variation in *P. radiata*, derived from 821 trees (113 endemic and 708 introduced) sampled across 16 countries. These include both highly domesticated material used in commercial forestry and genetically unaltered individuals from endemic populations. Genotyping was performed using genotype-by-sequencing (GBS), also known as double-digest RAD sequencing (ddRADseq), from *P. radiata* needle-derived genomic DNA. Alongside the SNP-by-sample dataset, we provide an identity-by-state (IBS) matrix capturing genetic relatedness among individuals. These resources can help trace the genetic origins of current tree breeding selections, identify opportunities to introgress novel genetic material to improve form, growth, and resilience, understand invasiveness of the taxon and guide efforts in conservation and restoration while maintaining the genetic integrity of endemic populations of this globally important species.

Specifications Table

**Table:** 

Subject	Biology
Specific subject area	*Pinus radiata, genotyping, genomics, forestry, conservation*
Data format	Filtered, processed.
Type of data	*.* TSV Table of SNP markers by sample (tabular form of .VCF) . CSV Table of sample metadata . CSV Resemblance matrix of Identity-by-State (Genetic Similarity) among samples
Data collection	Needles were collected from 821 individual *P. radiata* trees at four cardinal points around the canopy. Within-tree samples were pooled and chopped into 1–3 mm fragments. DNA from each sampled tree was extracted in quadruplicate using the Qiagen DNeasy Plant Pro kit and then pooled. Genotype-by-sequencing (GBS) used a dual restriction-enzyme system based on *EcoR1* and *Mse1*. Sequencing was conducted across five lanes of a NovaSeq 6000 platform (SP flow cell, 300 cycles per lane) at the Australian Genome Research Facility, Sydney, Australia.
Data source location	Locations spanned 16 countries including all five endemic populations (Isla Cedros and Isla Guadalupe, Baja California Mexico, and Monterey, Cambria, and Año Nuevo, California USA) and introduced global plantings (Argentina, Australia, New Zealand, Sweden, Chile, Portugal, Spain, France, USA, China, India, England, Ireland, South Africa and Greece). Each sample was GPS marked; latitude and longitude values (geographic coordinate points) are included in the primary sample metadata table.
Data accessibility	Repository name: Dryad data repository Data identification number: DOI: 10.5061 Direct URL to data: https://doi.org/10.5061/dryad.bzkh189pc
Related research article	None

## 1 Value of the Data

• The dataset provides information on the genetic relatedness of globally distributed *Pinus radiata* populations. These include genetically conserved ancestral populations from the species’ native range (Isla Cedros and Isla Guadalupe, Baja California Mexico, and Monterey, Cambria, and Año Nuevo, California USA), alongside globally introduced populations that have undergone selective breeding and domestication to various extents.
• *Pinus radiata* is one of the world’s most widely grown plantation tree species. Information on the genetic structure of *P. radiata* populations provide important information for how domestication has shaped genetic variation to date, including source tracking and reconstruction of origin and spread from the endemic populations. *Pinus radiata* is also a widespread invasive species, especially in Mediterranean climate regions. These data can thus also be useful to understand invasiveness of the taxon. The dataset may provide information on founder effects and where new sources of allelic variation can be integrated into future tree breeding efforts such as identifying adaptive traits and guide in the selection of resilient genotypes.
• Native populations of *P. radiata* are endangered. GBS data can assist conservation by helping ensure that the native populations retain their unique genetic identity. This supports the preservation of traits related to local adaptation, prevents genetic homogenization with other populations, and avoids the loss of rare alleles important for long-term resilience.
• *Pinus radiata* holds significance for some Indigenous communities as part of culturally and ecologically important landscapes. These data may aid in preserving the genetic integrity of local populations, supporting indigenous-led restoration and conservation efforts that aim to avoid extirpation of populations, restore habitats, and maintain both the species and the ecosystems they are embedded within.

## 2 Background

*Pinus radiata* is naturally restricted to five native populations—three along California’s coast and two on Mexican islands [1,2]. Within this native range, it is classified as ‘endangered’ by the IUCN due to disease, herbivory, fire, and climate stress [3]. Restoration efforts are underway, with a key focus on understanding genetic structure within and among native populations to ensure conservation actions preserve local provenance and species identity.

Despite its endangered status, *P. radiata* has become the most widely cultivated conifer globally, thriving in plantations across Australia, New Zealand, Chile, and beyond [4]. All global plantings trace back to the five native populations, though the extent of genetic admixture, selection, and domestication varies by region [5]. Comparing cultivated trees with native progenitors helps identify untapped genetic variation, including divergence, introgression, and ancestral allele recession. This is vital for breeding programmes aiming to improve resistance to pests, enhance silvicultural traits, and support climate resilience.

We present a new dataset describing genetic relatedness among 821 *P. radiata* individuals, representing both native and introduced populations. Generated using GBS, this dataset is a valuable resource for studies on genetic structure, diversity, and provenance, supporting efforts in conservation, forest management, and breeding.

## 3 Data Description

**Fig. 1:**
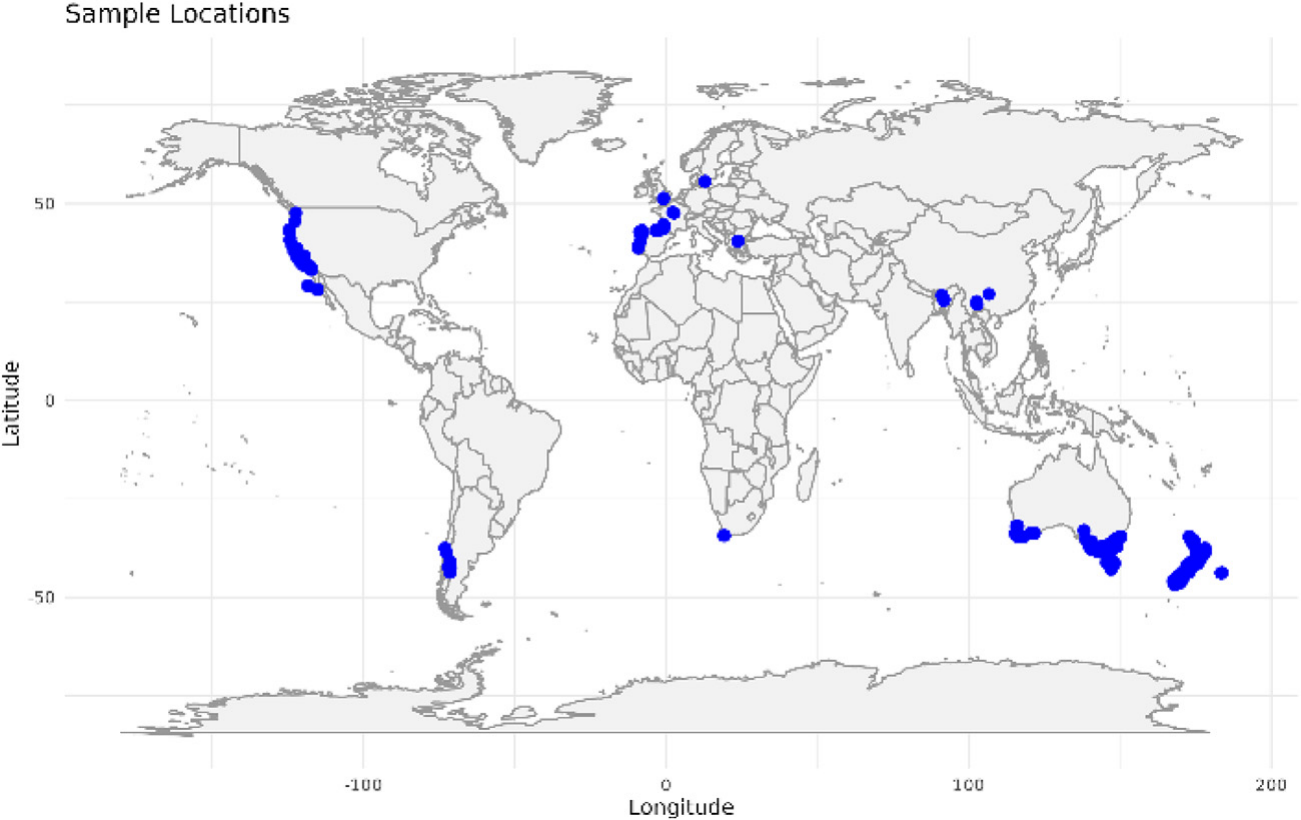
Locations of sampled *Pinus radiata* across 16 countries to generate the genotype-by-sequencing dataset.

The dataset comprises genotype-by-sequencing information collected for 821 *Pinus radiata* trees (113 native and 708 introduced) that were sampled across 16 countries (Fig. 1). These data are held in a Dryad data repository [6], and comprise:

(1) a main data table of SNPs (genotypes) for all samples
(2) identity-by-state genetic similarity matrix derived from the SNP data table
(3) the metadata including geo-reference location points of each sample, endemic status and sampling country.

## 4 Experimental Design, Materials and Methods

### 4.1 Systematic sampling

We used a combination of dedicated sampling and community science to collect *Pinus radiata* needle (leaf) tissue for DNA extraction and genotyping. The native *P. radiata* populations have evolved in geographic isolation and are genetically distinct and vary in adaptive traits. We therefore specifically targeted these populations to ensure the capturing of as much ancestral genetic variation as possible within the species. For samples of *P. radiata* in the introduced global range, we sought assistance from research groups to collect needle samples and extract DNA from them. The full extent of the locations sampled is shown in Fig. 1. A full metadata list of sample names with locations including GPS co-ordinates, country and introduced or endemic can be found in [6].

To enable consistency in handling and processing, DNA extraction, purification and shipping, we provided detailed standard operating procedures, information via email communication, and walk-throughs on a YouTube channel (https://www.youtube.com/@treemicrobiome). Furthermore, a bespoke bilingual smartphone application (PineSpy) guided tree identification and sampling, generated unique IDs for chain-of-custody tracking, confirmed user permissions, and recorded location metadata.

Needles were collected from the four cardinal points around individual *P. radiata* tree crown perimeters. These were chilled for transport to the laboratory. In sterile environments, the needles of each individual tree were pooled into a single composite sample and chopped using EtOH sterilized scissors into 1–3 mm fragments. Genomic DNA (gDNA) was extracted from approximately 50 mg of each sample using the Qiagen DNeasy Plant Pro kit. Extractions were conducted in quadruplicate to both mitigate against the possibility an individual extraction failed, and so that pooled extractions would contain more final and representative gDNA than a sole extraction.

Purified gDNA was genotyped using genotype-by-sequencing (GBS) [7], otherwise known as ‘double digest restriction-site associated DNA sequencing’ or ddRADseq. While SNP chip-based tools are available for *P. radiata* [8], GBS was used as it identifies thousands of *de novo* SNPs which is preferable when working across diverse or poorly characterized populations (e.g. wild and domesticated *P. radiata*). As SNP chips are mostly suited for assessing *a priori* targeted variable sites, application to poorly studied populations can be unsuitable, missing potentially important variation. GBS can capture both rare and population specific variants, aiding the analysis of population genetic structure [7]. Additionally, the ability to multiplex hundreds of samples in a single sequencing run makes GBS a cost-effective option for large-scale genotyping.

DNA was digested using a dual-enzyme approach with *EcoRI* and *MseI*, and custom barcoded adapters compatible with the restriction site overhangs were ligated to the resulting fragments. Fragments in the 280–375 bp size range were selected using BluePippin automated gel electrophoresis. Sequencing was carried out across five lanes of an Illumina NovaSeq 6000 (SP flow cell, 300 cycles per lane).

Sequencing generated a total of 1.63 billion paired-end reads (489.01 Gb) across four libraries, with per-library yields ranging from 362 to 464 million reads (109–139 Gb). On average, each sample produced 5.19 million clustered reads, with a clustering efficiency of 73.8 %, indicating that nearly three-quarters of raw reads were retained after demultiplexing and quality filtering. These values demonstrate sufficient sequencing depth and read retention to ensure robust SNP discovery and reliable downstream analyses.

Raw sequencing reads in pooled fastq.gz files were demultiplexed and processed using a de novo k-mer–based clustering and genotyping pipeline [9]. A k-mer size of 31 bp was used to identify quasi-exact read matches and organize them into clusters. This process began with the NSGEP algorithm assigning the first read to initiate the first cluster. Each subsequent read was compared to existing cluster consensus sequences. If a read's representative k-mer was sufficiently similar (within 1 bp difference) to a cluster’s consensus, it was added to that cluster; otherwise, a new cluster was formed. To avoid bias near restriction enzyme cut sites, representative k-mers were extracted beginning at the sixth base from the 5′ end of each read. This k-mer was used to determine similarity between reads and update consensus sequences dynamically. Reads with up to 2 bp variation in the k-mer region could be clustered together if both shared a 1 bp difference with the evolving consensus. Singleton clusters were removed to reduce noise.

After clustering, the full set of reads was scanned a second time to assign them to their respective clusters. Unassigned reads were discarded. Clusters were then processed in parallel. The consensus sequence for each cluster was extended to match the length of the longest read in that cluster. For paired-end reads, forward and reverse reads were aligned and merged to form complete consensus sequences. These consensus sequences were used as references, resulting in a Variant Call Format (VCF) file containing the final variant data. These data were filtered and a final genotype x sample file with sample name and location metadata added to generate an ‘analysis friendly’ datafile [6].

Across 1597,896 SNP loci genotyped, the median per-sample call rate was 77.1 % (average 65.9 %). Per-SNP and per-individual filtering thresholds were applied to retain high-quality data: SNPs with ≥90 % call rate, individuals with ≤10 % missing data, and SNPs with minor allele frequency ≥0.05 were kept for downstream population genetic analyses.

**Fig. 2:**
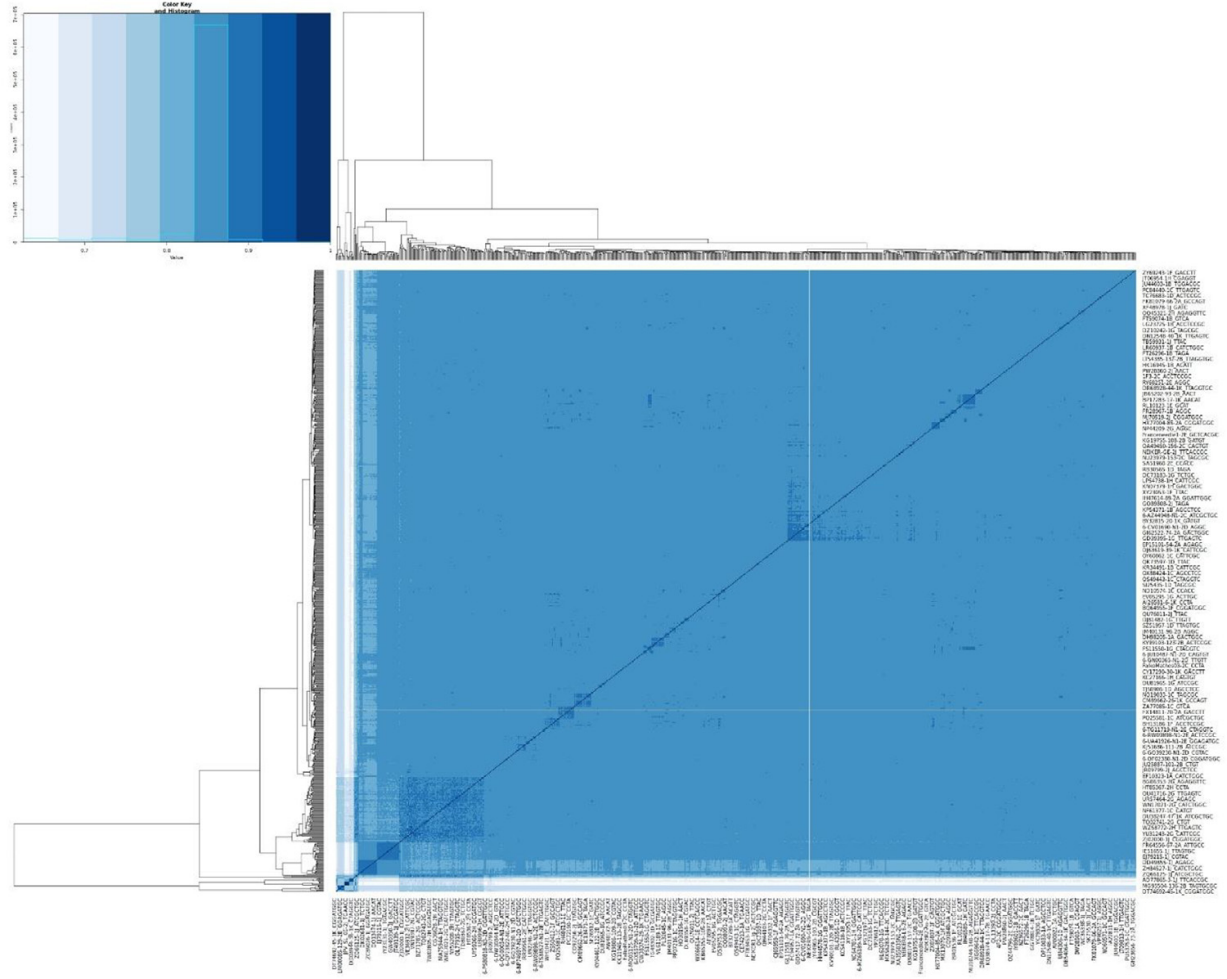
Heatmap and clustering of identity-by-state (IBS). On the 'Colour Key and Histogram' figure at the top left corner IBS value equal 1 means 100 % identity across genotypes in tested loci for pairs of compared samples (the darkest colour on heat map). The most distinct samples show the lowest IBS score (the lightest colour on the heatmap). Samples are ordered according to similarity calculated by the hclust algorithm. This algorithm uses pair-wise IBS scores as a distance matrix to produce a dendrogram plot.

**Fig. 3:**
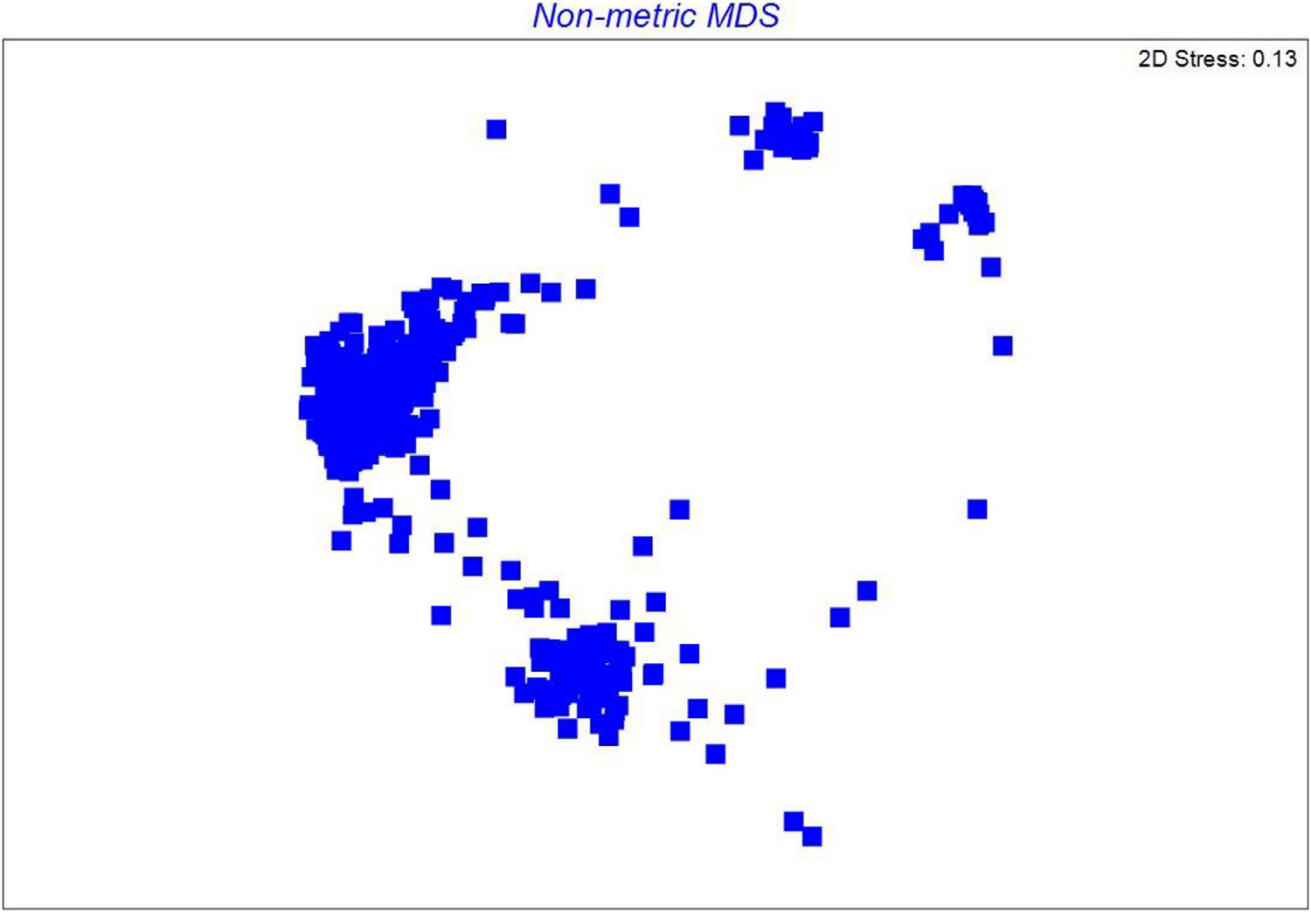
Non-metric multidimensional scaling of the identity-by-state similarity matrix.

The PLINK software [10] was used to derive pairwise identity-by-state (IBS) values between all individuals from the SNP data. This allows quantification of genetic similarity between individual samples, i.e. the proportion of shared alleles [6]. Fig. 2 depicts a heatmap and clustering of identity by state (IBS). This plot is illustrating the pairwise similarity across samples and allows quick detection of sample groups with close genotypes. The pairwise genetic similarity matrix was analysed using non-metric multidimensional scaling (nMDS) in PRIMER [11] to examine overall patterns of population structure (Fig. 3).

## Limitations

None.

## Ethics Statement

The authors have read, and follow the ethical requirements for publication in Data in Brief and confirming that the current work does not involve human subjects, animal experiments, or any data collected from social media platforms.

## Credit Author Statement

**Sarah Addison**: Methodology, Validation and Data Curation, Writing - original draft, Writing - review & editing. **Luciana L. Mendoza:** Conceptualization, Writing - review & editing, **Megan A. Rúa:** Conceptualization, Writing - review & editing**, Peter Clinton**: Conceptualization, Writing - review & editing, **Global Tree Root Microbiome Consortium:** Methodology, Writing – review & editing. **Steve Wakelin**: Conceptualization, Methodology, Writing - original draft, Writing - review & editing.

**Global Tree Root Microbiome Consortium Author List**

Dimitrios Avtzis⁶, Mark R. Bakker⁷, Tiara Barriga-Barra⁸, Vanina Benoit⁹, Frédéric Bernier¹⁰, Helena Bragança¹¹^,^¹², Nicolas Cobo⁸, Pierre-Emmanuel Courty¹³, Kaitlyn Daley¹, Barbara Doyle Prestwich¹⁴, Nina Eichler¹, Joan Antoni Estades¹⁵, Mariangela Fotelli⁶, Alain Gardiennet¹³, Sahar Gharabaghlou¹⁶, Madeline R. Greene⁴, Anna Harris¹⁷, Javier Herrero¹⁵, Isabel Henriques¹⁸^,^¹⁹, Emma Hutchinson¹⁴, Dean Irving²⁰, Lewis Jewell¹⁷, Victoria Knight¹⁴, Margarita Lema²¹, Frederico Leitão¹⁸^,^¹⁹, Eoin Lettice¹⁴, Johannes J. Le Roux²²^,^²³, Falko Mathes²⁴, Lucía Molina²⁵^,^²⁶, Itumeleng Moroenyane¹⁶, Arpan Mukherjee²⁷, Claire Nolan¹⁴, Ida Nordström²⁸, Maria Belen Pildain²⁵^,^²⁶, Glória Pinto¹⁸, Jay Prakash Verma²⁷, Ana Silva¹¹^,^¹², Mariah Slaughter⁴, Simeon Smaill⁵, Marjorie Santamaria¹⁴, Sarah J. Sapsford²^9^, Sabai Shwe¹, Roberto Touza²¹, Rodrigo Vargas-Gaete⁸^,30^, Louise V édrenne¹³, Yanliang Wang³^1^, Zhen-Zhen Yan³^2,^³^3^, Fuqiang Yu³^1^, Jing Yuan³^1^, Mnqobi Zuma²⁸.

^6^ Forest Research Institute, Hellenic Agricultural Organization Dimitra, Vassilika, 57,006 Thessaloniki, Greece.

^7^INRAE, Bordeaux Sciences Agro, ISPA, Villenave d’Ornon, France.

^8^ Facultad de Ciencias Agropecuarias y Medioambiente, Universidad de La Fontera, Chile.

^9^ INRAE, ONF, BioForA, F-45,075 Orléans, France.

^10^ INRAE, route d’Arcachon 69, Cestas, France.

^11^ INIAV I. P. Instituto Nacional de Investigação Agrária e Veterinária, I.P., Quinta do Marquês, 2780–159 Oeiras, Portugal.

^12^ GREEN-IT Bioresources for Sustainability, ITQB NOVA, *Av*. da República, 2780–157 Oeiras, Portugal.

^13^ Agroécologie, AgroSup Dijon, CNRS, Université de Bourgogne, INRAE, Université de Bourgogne Franche-Comté, Dijon, France.

^14^ School of Biological, Earth and Environmental Science and Environmental Research Institute, University College Cork, Cork, Ireland.

^15^ NEIKER - Basque Institute for Agricultural Research and Development - Basque Research and Technology Alliance (BRTA). Campus Agroalimentario de Arkaute s/n, 01,192, Arkaute, Spain.

^16^ Department of Botany and Zoology, Stellenbosch University, South Africa.

^17^ Forest Research, Alice Holt Lodge, Farnham, UK.

^18^ Centre for Environmental and Marine Studies (CESAM), Department of Biology, University of Aveiro, Campus Universitário de Santiago, Aveiro 3810–193, Portugal.

^19^ Centre for Functional Ecology, Associate Laboratory TERRA, Department of Life Sciences, Faculty of Sciences and Technology, University of Coimbra, 3000–456, Coimbra, Portugal.

^20^ Forest Products Commission, Perth, Western Australia, Australia.

^21^ Misión Biológica de Galicia. Consejo Superior de Investigaciones Científicas. Pontevedra, Apdo 28, Pontevedra 36,080, Spain.

^22^ School of Natural Sciences, Macquarie University, North Ryde 2113, New South Wales, Australia.

^23^ Centre for Invasion Biology, Department of Botany and Zoology, Stellenbosch University, Stellenbosch 7600, South Africa

^24^ Commonwealth Scientific and Industrial Research Organisation, Environment, Waterford, Western Australia 6152, Australia

^25^ Área de Fitopatología y Microbiología Aplicada, Centro de Investigación y Extensión Forestal Andino Patagónico (CIEFAP), Esquel, Chubut, Argentina.

^26^ National Scientific and Technical Research Council (CONICET), Argentina.

^27^ Plant-Microbe Interaction Lab, Institute of Environment and Sustainable Development (IESD), Banaras Hindu University, Varanasi-221,005, Uttar Pradesh, India.

^28^ Southern Swedish Forest Research Centre, Swedish University of Agricultural Sciences, Box 190, 234 22, Lomma, Sweden.

^29^ Harry Butler Institute, Murdoch University, Murdoch, Western Australia, 6150, Australia.

^30^ Facultad de Ciencias Agropecuarias y Medioambiente, Universidad de La Fontera, Chile.

^31^ The Germplasm Bank of Wild Species & Yunnan Key Laboratory for Fungal Diversity and Green Development, Kunming Institute of Botany, Chinese Academy of Sciences, Kunming, Yunnan, 650,201, China.

^32^ Australian Rivers Institution and School of Environment and Science, Griffith University, Nathan Campus, QLD, 4111, Australia.

^33^ Hawkesbury Institute for the Environment, Western Sydney University, Hawkesbury Campus, Penrith, NSW, Sydney, Australia; Global Centre for Land-Based Innovation, Western Sydney University, Hawkesbury Campus, Penrith, NSW, Sydney, Australia.

Funding for this work, which sits under ‘C04X2002 The Tree Microbiome Project: at the root of climate proofing forests’, was from the New Zealand Ministry of Business, Innovation and Employment (MBIE) and the Forest Growers Levy Trust.

## Data Availability

Mendeley DataA genotype-by-sequencing dataset and identity-by-state matrix of genetic variation in Pinus radiata from 16 counties (Original data) (Dryad).
